# Radiation for MALT of the Submandibular Gland

**DOI:** 10.1155/2017/8397621

**Published:** 2017-01-09

**Authors:** Juskaran Chadha, Marita S. Teng, Julie Teruya-feldstein, Richard L. Bakst

**Affiliations:** ^1^Department of Medicine, Mount Sinai West, 1000 Tenth Avenue, New York, NY 10019, USA; ^2^Department of Otolaryngology, Icahn School of Medicine at Mount Sinai Hospital, New York, NY 10029, USA; ^3^Department of Pathology, Icahn School of Medicine at Mount Sinai Hospital, New York, NY 10029, USA; ^4^Department of Radiation Oncology, Icahn School of Medicine at Mount Sinai Hospital, New York, NY 10029, USA

## Abstract

We are reporting a case of a 27-year-old woman with a history of swelling in the left submandibular region. This swelling was associated with a mass, and this was pathologically confirmed to be an extranodal marginal zone lymphoma (MALT). The patient underwent surgical excision and postoperative adjuvant radiation therapy. The patient tolerated treatments well and remains free of disease. Here, we describe the case and management described in the current literature.

## 1. Introduction

Non-Hodgkin lymphoma (NHL) is the sixth most commonly diagnosed cancer in both men and women in the United States. In 2015, there were an estimated 72,580 new cases of NHL [1]. Clinicopathological features and immunophenotyping have helped classify several subtypes of NHL. Mucosa-associated lymphoid tissue (MALT) lymphoma arises from B cells and is a type of extranodal marginal zone lymphoma [2]. The incidence of lymphoma of the MALT type is approximately 5% [1, 2]. In the US 2001–2009 SEER database, Khalil et al. [3] identified 8,821 cases of extranodal marginal zone lymphoma. The incidence rates (IR) noted stomach to be the most common site (IR 3.8), followed by spleen (IR 1.6), eye/adnexa (IR 1.4), and lung, skin, and salivary glands (IRs 0.9–1.0). In a population-based study from 1994 to 2009, Vazquez et al. [4] noted 0.086 cases/100,000 individuals of MALT lymphoma arising from the salivary gland. Similar to other reports in the literature, the most common major salivary gland involved was the parotid (80.9%).

Overall, submandibular gland involvement is exceedingly rare. We report a case of primary MALT lymphoma of the submandibular salivary gland and review of literature on treatment of this clinical presentation.

## 2. Case Report

**Figure 1:**
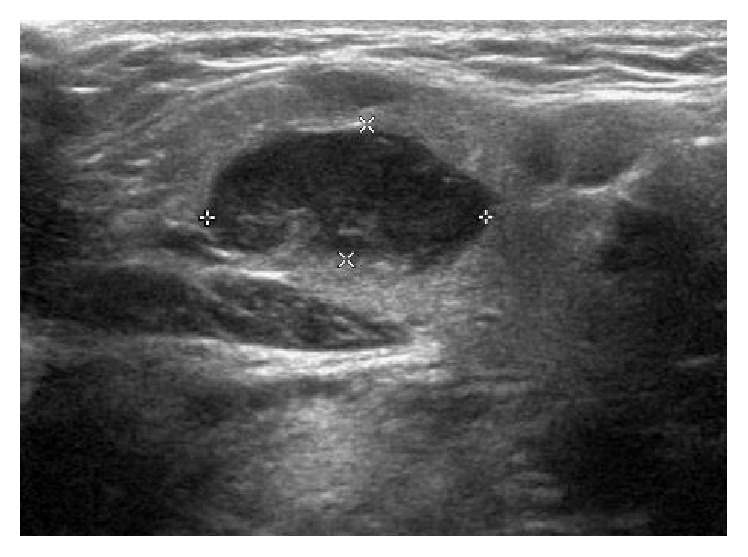
Diagnostic ultrasound of the left submandibular gland and neck.

**Figure 2:**
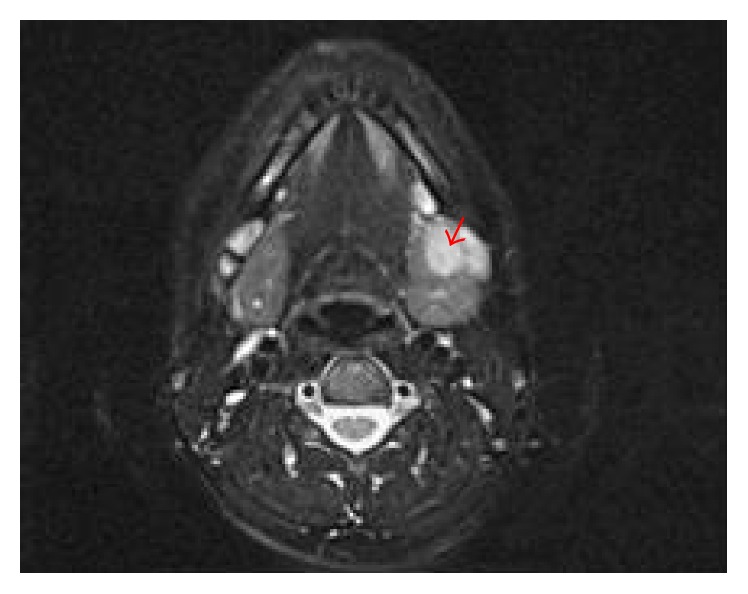
Axial T2 MRI of the head and neck at initial presentation illustrating the left submandibular gland mass (arrowhead).

The patient is a 27-year-old woman who presented with history of swelling in the left submandibular region. She was otherwise asymptomatic and denied fevers, chills, night sweats, fatigue, and weight loss. Ultrasound of the swelling on the left side of her neck reported a 1.9 × 0.9 × 0.9 cm hypoechoic mass involving the left submandibular gland (Figure 1). MRI of the neck revealed a 1.4 × 1.1 × 1.9 cm enhancing lesion within the left submandibular gland near the anterior aspect of the gland (Figure 2). Initial FNA of the left submandibular gland showed reactive cells, and core needle biopsy revealed dense lymphoid infiltrate without enough tissue to make a definitive diagnosis.

**Figure 3:**
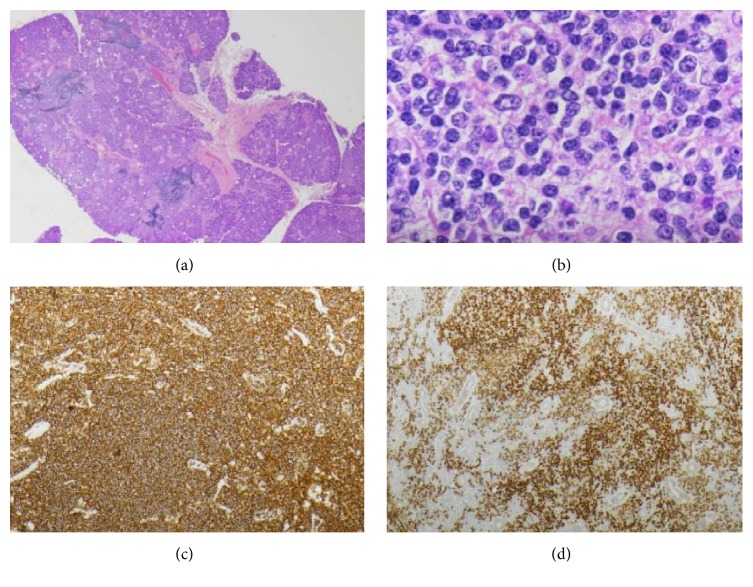
Pathology demonstrated moderate to marked atypical small lymphoid proliferation, involving the parotid gland parenchyma and ducts, composed of predominantly small lymphoid cells on low-power (a) and high-power (b) H&E. Immunohistochemistry demonstrated that the B cells were positive for CD20 (c) and PAX 5 (d), consistent with a MALT.

The patient underwent submandibular gland excision. Pathology showed moderate to marked atypical small lymphoid proliferation, and immunohistochemistry revealed moderate to marked lymphoid expansion. The B cells were positive for CD20, PAX 5, and CD79a, with coexpression of CD43, BCL-2, prominent CD23, and positive dendritic network, while they were negative for CD3, CD5, CD10, MUM1, BCL-6, cyclin D1, and CD23, with a low proliferative fraction of 10–20%. Based on these pathology findings, the diagnosis of extranodal marginal zone lymphoma of MALT type in the submandibular gland was established (Figure 3). Bone marrow biopsy was found to be negative. PET/CT revealed increased uptake in the submandibular gland and showed no evidence of hypermetabolic lymphadenopathy above or below the diaphragm.

**Figure 4:**
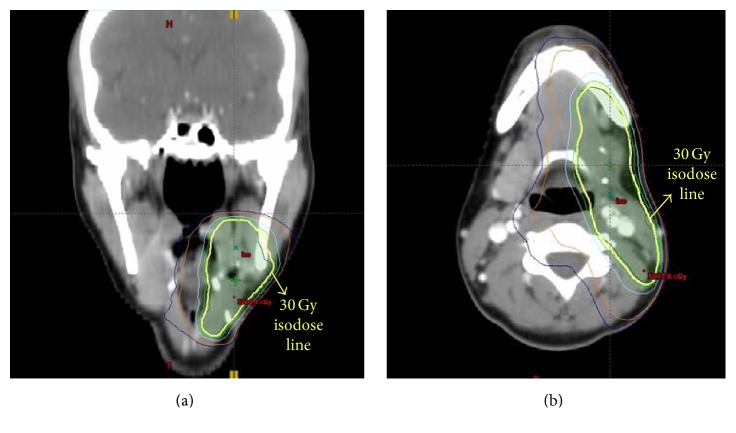
Coronal (a) and axial (b) images of radiation therapy treatment IMRT plan to 30 Gy.

Following the excision, the patient presented to our department for consideration of radiation. The patient received radiation to the left submandibular gland surgical bed and adjacent draining lymph nodes using IMRT (Figure 4) to 30 Gy in 15 fractions. The patient tolerated radiation very well and only reported mild dysgeusia during the third week of treatment, which resolved 1 month following treatment. At her 10-month follow-up, the patient's saliva has returned to baseline and she remains without evidence of disease on repeat PET/CT.

## 3. Discussion

The etiology of lymphoma is not well understood; however, there are a number of risk factors associated with higher incidence of lymphoma. Risk factors associated with salivary gland MALT lymphoma include chronic immune system dysregulation such as Sjögren's syndrome [5–8] and chronic inflammatory conditions such as sialadenitis [9–11]. In a large multicenter international study on MALT lymphoma of the salivary gland, 40% of patients had history of autoimmune disease [12]. In the Rare Cancer Network multicenter study [13], 28% of patients had documented history of sialadenitis. Our patient did not have any known risk factors.

Table 1 is a summary of published series on salivary gland lymphoma. The International External Lymphoma Study Group (IELSG) reported on 247 patients with MALT lymphoma of the salivary glands [12]. In this study, only 5% had primary submandibular gland involvement; in the majority (78%), the parotid gland was involved. 59% of patients presented with limited stage. Local therapy, chemotherapy, and palliative therapy were delivered to 57%, 37%, and 6% of patients, respectively. The median overall survival and progression-free survival (PFS) were 18.3 years and 9.3 years, respectively. On multivariate analysis, age < 60 years and low to intermediate International Prognostic Index were associated with improved PFS.

Anacak et al. [13] reported on 63 patients diagnosed with MALT lymphoma of the salivary glands from the 13 member centers of the Rare Cancer Network from 10 countries. The involved site at presentation included 49 patients with parotid, 15 patients with submandibular gland, and 3 patients with minor glands involved. Multiple glands were involved in 9 patients. The median age was 58 years; 47 patients were female and 16 were male. Approximately 70% of patients had stage IE disease. In this series, the majority of patients received surgery plus RT (*n* = 23). The median RT dose delivered was 36 Gy. Importantly, RT significantly reduced the rate of relapse, as they observed recurrence rates of 26.8% (11/41) in patients receiving RT and 57% (12/21) in patients not receiving RT (*p* = 0.027). Specifically, the use of RT was associated with improved 5-year disease-free survivals (DFS); higher rates of DFS were observed with RT (66.1%) compared to when no RT (34.4%) was delivered (*p* < 0.01). Other factors influencing disease-free survival were stage and residual disease (*p* < 0.01).

The various treatment options for localized MALT lymphoma include observation, surgery, RT, or combination of surgery plus RT and/or systemic therapy.  Table 2 is a summary of case reports published on lymphoma of the submandibular salivary glands. In the literature, there is variability in the type of treatments delivered. This perhaps reflects the retrospective and multicenter nature of case reports, as well as the lack of consensus for treating this less commonly seen presentation of MALT lymphoma.

Teckie et al. [14] reported on long-term outcomes and patterns of relapse for early-stage extranodal marginal zone lymphoma treated with primary RT. They studied 490 consecutive patients with stage IE or IIE marginal zone lymphoma between 1992 and 2012. Of those, 244 patients received RT alone. The most common site of disease was the stomach (50%); other sites included orbit (18%), nonthyroid head and neck (8%), skin (8%), and breast (5%). The majority (92%) of patients had stage IE, and the median RT dose delivered was 30 Gy. On multivariable analysis, the factors significantly associated with RFS included the primary disease site (*p* = 0.007), age (*p* = 0.04), presence of B-symptoms (*p* = 0.02), and International Prognostic Index risk group (*p* = 0.03). Among all sites of MALT lymphoma presentation, head and neck and gastric sites had better relapse-free survival. In this large cohort of 244 patients, higher rates of overall and cause-specific survivals were observed when treated with curative RT alone.

Tsang et al. [15] studied 103 patients with localized stages IE and IIE MALT lymphoma. Majority of patients (*n* = 85) received RT alone, and a median dose of 30 Gy was delivered. The crude local control rate with RT was 95.3% (81 of 85 patients). They observed excellent local control rate with moderate-dose RT and suggested a curative potential for three-fourths of the patients. Another retrospective study reported on 77 consecutive patients with stages I and II MALT lymphoma. This group included 52 patients (68%) treated with local RT alone, 17 (22%) undergoing surgery followed by adjuvant RT, 5 (6%) having surgery alone, 2 (3%) undergoing surgery and chemotherapy, and one patient receiving chemotherapy alone [16]. The median RT dose was 30 Gy (range: 18–40 Gy). They reported significantly better results with RT. Improved 5-year progression-free survival rate (79% versus 50%; *p* = 0.002) and freedom from treatment failure rate (81% versus 50%; *p* = 0.0004) were noted in patients who received RT as compared to those who did not.

Long-term outcomes for patients with early-stage extranodal marginal zone lymphoma (EMZL) and mantle cell lymphoma were recently reported in a paper by Barzenje et al. [17]. Their study included 49 patients with EMZL. Radiotherapy was used in 40 patients and surgery alone in 9 patients. The distribution by site noted that 13/40 patients receiving RT alone had salivary gland involvement. Overall, this paper observed that radiotherapy alone was effective in treating EMZL, with low relapse rates and a 10-year survival of 78%. In a phase II study, 37 patients with stage IE extragastric MALT lymphomas including orbit, salivary gland, and thyroid were treated with RT [18]. The median RT dose delivered was 30.6 Gy. They observed 97.3% local control and 3-year progression-free survival of 91.9% and concluded that moderate-dose RT is highly effective in achieving local control with acceptable morbidity.

Other series have also observed long-lasting remissions and likely cures using RT. Goda et al. [19] reported long-term outcomes on 167 patients with stages I and II MALT lymphoma who received RT. This series included 28 patients with salivary glands as the site of presentation. The median RT dose delivered was 30 Gy. For patients with primary salivary gland presentation, the 10-year relapse-free rate was 68%. They observed minimal toxicity with moderate-dose RT.

Treatment guidelines by the International Lymphoma Radiation Oncology Group recommend involved-field RT [20]. Specifically, for salivary glands, the recommendation is to include the whole unilateral involved gland and any adjacent concerning node using conformal planning techniques. Distant relapse is rarely observed in patients treated with RT as the sole treatment modality [14, 19]. These data suggest that, for early-stage disease, involved-field RT is the preferred approach. Our patient was treated with involved site RT to a total dose of 30 Gy and remains without evidence of disease at 10-month follow-up and with minimal associated toxicity.

In summary, the optimal treatment of salivary gland MALT lymphomas has not been clearly defined. Patients with MALT lymphoma generally present with early-stage disease and have favorable outcomes. Given that patients survive for many years, side effects and long-term complications of therapy are a critical consideration when recommending initial treatment. For localized disease, conservative excision and adjuvant RT represent a reasonable treatment option with excellent response, local control, and minimal toxicity. Patients are spared the morbidity associated with more extensive salivary gland surgery, and radiation therapy has an important role in the curative treatment of localized MALT lymphoma.

## Figures and Tables

**Table 1:** Summary of published series on salivary gland lymphoma.

Study	Number of cases	Gender ratio	Age range at diagnosis	Path	Associated conditions	Stage	Treatment
Jackson et al., 2015 [12]	247	M : F 1 : 3	18–93 years	MALT	Autoimmune disorder, 41%	76% Localized disease	Surgery, RT or both, 57% Systemic therapy, 37% Observed, 6%

Anacak et al., 2012 [13]	63	M : F 1 : 3	26–92 years	MALT	Sialadenitis, 28% Sjögren's syndrome, 6% Cysts, 13% Calcifications, 16%	73% Stages I-II	Surgery, RT or both, 63.5% Systemic therapy ± surgery ± RT, 34.8% Supportive care, 1.6%

MALT: mucosa-associated lymphoid tissue.

**Table 2:** Summary of case reports on submandibular salivary gland lymphoma.

Author year	Number of cases	Gender	Age at diagnosis	Path	Associated conditions	Stage	Treatment
Hyman and Wolff, 1976 [21]	2	M	38	Lymphocytic		III	Surgery + RT
		F	73	Nodular		II	RT bilateral neck

Gleeson et al., 1986 [22]	5	M : F^*∗*^ 1.5 : 1	Range: 50–70^*∗*^ years	FMx	Sicca^*∗*^ syndrome in some	I	Surgery
				DSC		I	Surgery + RT
				FSC		II	Surgery + RT + chlorambucil
				DSL		II	Biopsy + RT + chlorambucil + surgery
				FSC		III	

Kojima et al., 2001 [23]	2	F	50	MALT	None	I	Observation
		F	64			I	Unknown

Ochoa, 2001 [9]	1	M	65	MALT	Chronic sclerosing sialadenitis		Surgery

Kojima, 2003 [10]	3	M	48	FL	Chronic pharyngitis	I	Surgery
		F	64		None	I	Surgery
		F	68		None	I	Surgery

Perera, 2010 [7]	1	F	73	MALT	Amyloidosis	I	Observation

Movahed et al., 2011 [6]	1	F	35	MALT	Sialadenitis	III	Observation

Shashidara et al., 2014 [24]	1	F	40	FL	None	I	Observation

Chen et al., 2016 [5]	1	F	24	MALT	Sjögren's syndrome	I	Surgery

Current report, 2016	1	F	27	MALT	None	I	Surgery + RT

MALT: mucosa-associated lymphoid tissue; FMx: follicular mixed small and large cell; DSC: diffuse small cell; FSC: follicular small cleaved cell; DSL: diffuse small lymphocytic; FL: follicular lymphoma.

^*∗*^All 30 cases (Gleeson).
